# Human milk small extracellular vesicles elicit changes in inflammatory response in infant human intestinal enteroids

**DOI:** 10.1038/s41598-026-59527-2

**Published:** 2026-06-26

**Authors:** Kacie D. Waiters, Anthony S. Peidl, Hailey A. McAndrew, Ashfia F. Khan, Grace Adeniyi-Ipadeola, Hephzibah Nwanosike, Sana Sharif, M. Funmi Babajide, Micah Castillo, Preethi H. Gunaratne, Samaneh Karami, Hoa Nguyen-Phuc, Xinli Liu, Sasirekha Ramani, Tasneem Bawa-Khalfe

**Affiliations:** 1 Center for Nuclear Receptors & Cell Signaling, Department of Biology & Biochemistry, University of Houston, Houston, TX 77204 USA; 2 Department of Molecular Virology and Microbiology, Baylor College of Medicine, Houston, TX 77030 USA; 3 Institute for Drug Education and Research, College of Pharmacy, University of Houston, Houston, TX 77204 USA; 4 UH-Sequencing & Gene Editing Core, Department of Biology & Biochemistry, University of Houston, Houston, TX 77204 USA

**Keywords:** Diseases, Gastroenterology, Immunology, Medical research

## Abstract

Human milk (HM) is the key nutritional source for infants and the most effective preventative approach against serious gastrointestinal inflammatory diseases like necrotizing enterocolitis (NEC) in preterm infants. Numerous studies in animal models indicate HM-derived small extracellular vesicles (HMEV) reduce severity of inflammation; however, data on human-specific models are limited. Isolated HMEV were characterized from early-HM (colostrum and transitional), mature-HM, and a combined, pasteurized donor bank-like HM sample (pool-); pool-HMEV retains a profile aligned with HMEV from both early- and mature-HM samples. Exposure to pool-HMEV initiates unique gene signatures associated with reduction of inflammation including NFkB-driven TNFα signaling in human intestinal enteroids (HIEs) established from neonates with intestinal atresia or NEC. Prior exposure of HIEs to pool-HMEV lowers the magnitude of EGTA- and TNF-induced barrier disruption. This foundational research demonstrates HMEVs cause transcriptional and functional changes to the human intestinal epithelium and will support future studies on HMEV-based therapeutics.

**Supplementary Information:**

The online version contains supplementary material available at https://doi.org/10.1038/s41598-026-59527-2.

## Introduction

Human milk (HM) is a vital nutritional source for a developing newborn infant and is protective against inflammatory diseases such as necrotizing enterocolitis (NEC). HM is enriched with small extracellular vesicles (sEV) that work together with macronutrients, micronutrients, and other bioactive molecules to regulate biological processes associated with infant development^1,2^.The 100–200 nm human milk–derived small extracellular vesicles (HMEVs) facilitate cell-cell communication to direct the health of both mother and child^3,4^. Functional cargo is actively sorted into HMEV from the cell of origin and includes critical regulatory proteins, RNA, and DNA. HMEV composition is highly dynamic and varies within an individual over time and between individuals^5–7^. This inherent variability in HMEV composition, however, presents a significant challenge to understanding their biological impact on infant health.

The effect of HMEVs on the gastrointestinal epithelium have primarily been explored using transformed laboratory cell lines and animal models. These studies have described profound effects of HMEVs on reducing inflammation, enhancing epithelial proliferation and improving barrier function^6,8–14^. HMEVs have been associated with reducing the incidence of NEC in rat pups and confer protection from oxidative-stress induced damage in neonatal rats and IEC-6 intestinal epithelial cells^8,9^. HMEVs have also been shown to reduce the replication of clinically important pathogens such as rotavirus and respiratory syncytial virus in vitro^15^. While these studies have provided valuable fundamental information, much less is known about the uptake and effect of HMEVs on the human infant intestinal epithelium.

Simulation studies with digestive enzymes and low pH that mimic infant physiology show degradation resilience of the HMEVs and their continued uptake into intestinal epithelial crypt-like cells from fetal human intestines^16–18^. Additionally, a recent study showed that HMEVs can be taken up by an infant ileal human intestinal enteroid (HIE)^19^; however, the biological effects of such uptake were not determined. HIEs are established from stem cells in intestinal surgical samples or biopsies. These non-transformed ex-vivo tissue cultures contain multiple different intestinal cell types and reflect the cellular heterogeneity of the intestinal epithelium. HIEs have been widely used to study gastrointestinal physiology and host-pathogen interactions. We recently demonstrated that HIEs established from infant intestinal surgical tissues are transcriptionally, morphologically and functionally distinct from adult stem-cell derived HIEs that are extensively used in biomedical research and are an age and developmental stage-appropriate model to study infant specific diseases and nutritional factors in human milk^20^.

In this study, we determined the effect of these HMEVs on preterm ileal HIEs as a biologically relevant model system for the infant gut. To model the standard clinical exposure of infants receiving donor bank human milk, and to establish a reproducible baseline across experiments, we utilized HMEVs isolated from pasteurized, pooled donor milk. We demonstrate that HMEV exposure initiates transcriptionally changes of infant HIE to impact innate immune response and barrier integrity. This work is significant in establishing infant HIEs as a model system to understand the biological effects of HMEVs from pooled donor HM on the infant gastrointestinal epithelium.>

## Results

### Characteristics and composition of HMEV based on lactation stages

**Fig. 1:**
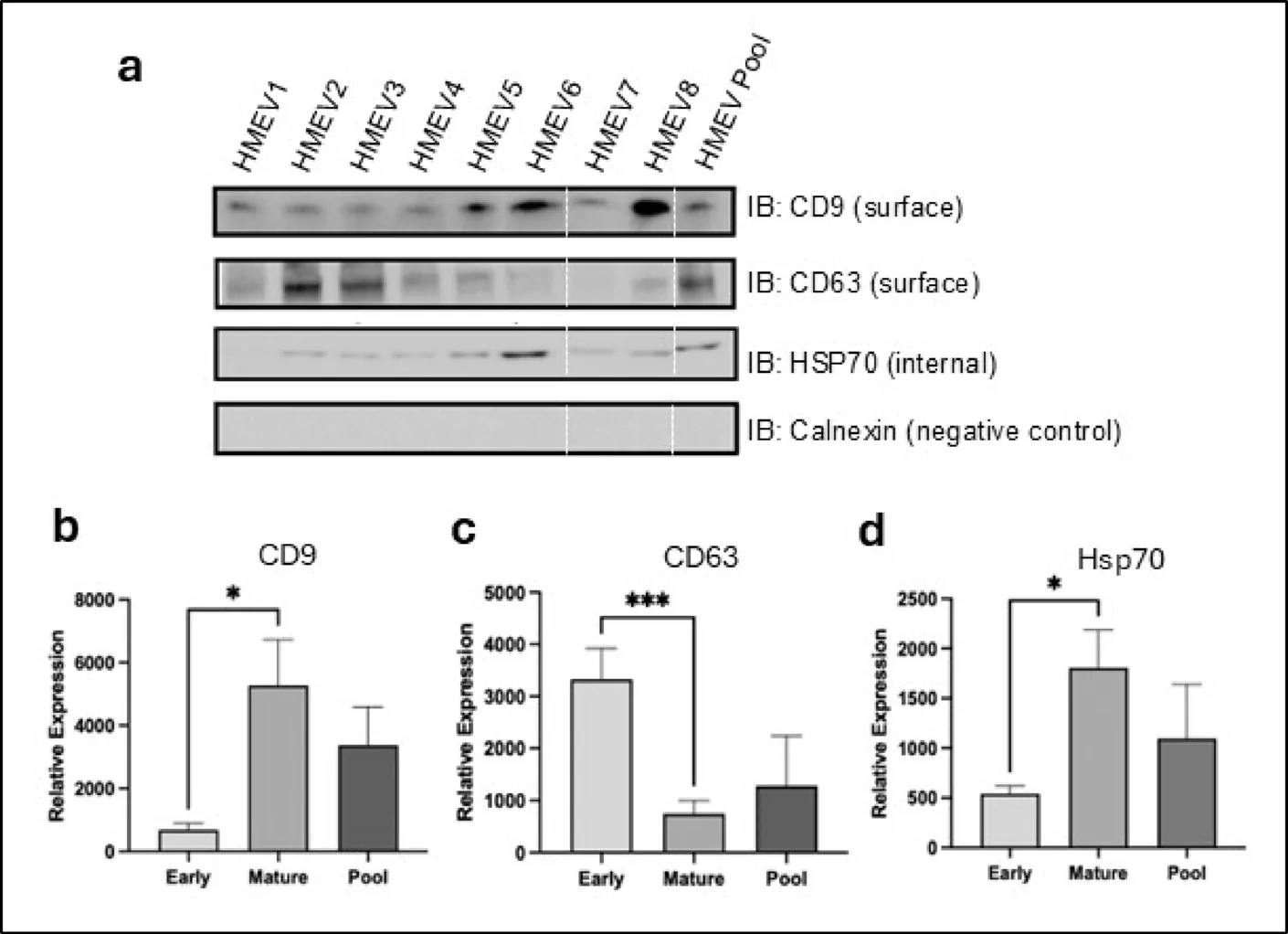
Exosomal Markers on Mature, Early, and Pool HMEV. Equal amounts of protein lysates (25 mg) from HMEVs harvested from individual human breast milk and the representative donor pool samples were subject to SDS-PAGE and immunoblotted for the presence of tetraspanin proteins CD9 and CD63, chaperone protein HSP70, and non-exosome isolated protein calnexin. (**A**). The blots are representative of two biological replicate experiments. Dashed lines indicate regions taken from the same blot; full, unedited blots are provided in the Supplementary Data. (**B**-**D**). Densitometry with ImageJ was used to quantify blot intensity of CD9, CD63 and Hsp70, respectively and subject to ANOVA and Tukey statistical analysis to determine difference between early, mature, and Pool-HMEV with *p-value < 0.05 and ***p-value < 0.001 shown.

As outlined by the International Society of Extracellular Vesicles (ISEV)^21^, HMEV enrichment was confirmed using several approaches including nanoparticle tracking analysis and protein biomarkers. Extracellular vesicles isolated from individual milk samples exhibit nanoparticle diameters ranging from 106.3 to 188.3 nm. The average vesicle size from individual samples (mean ± SEM = 147.9 ± 5.6, Supplementary Fig. S1A) is similar to that of vesicles from the pasteurized pooled sample (Supplementary Fig. S1A-B) and is consistent with the definition of sEV with diameters of 30 to 200 nm^22^. Each exosomal preparation yielded an average vesicle concentration ranging from 0.54 × 10^12^mL to 9.34 × 10^12^/mL consistent with the concentration ranges obtained in a previous study^23^. However, we noted significantly lower concentrations of sEV from mature versus early HM samples but not as compared to the Pool sample (ANOVA and Tukey’s post hoc, *p* < 0.05, Supplementary Fig. S1C). We asked if differences in the sEV concentrations are associated with differences in biomolecule composition of the isolated HMEV. Inverse to the HMEV concentration, total RNA concentrations are significantly higher in HMEV isolated from mature HM as compared to early HM samples (ANOVA and Tukey’s post hoc, *p* < 0.05, Supplementary Fig. S2A). In contrast, HMEV DNA levels are slightly higher, but not statistically significant, in early versus mature HMEV (Supplementary Fig. S2B). Similarly, protein concentrations in HMEV from early HM samples are significantly higher than HMEVs from mature milk (ANOVA and Tukey’s post hoc, *p* < 0.05, Supplementary Fig. S2C). Proteins in HMEV includes exosomal surface tetraspanins (CD9 and CD63) and intra-exosomal Hsp70 but lacked non-exosomal calnexin protein (Fig. 1A). These protein biomarkers confirm exosomal enrichment and concurrently demonstrate lactation stage-dependent inclusion of exosomal proteins. Specifically, CD63 and CD9 exhibit inverse enrichment with greater CD63 in early HMEV (Fig. 1B) and CD9 enrichment in mature HMEV (Fig. 1C). Similarly, intra-exosomal marker Hsp70 is also higher in mature than early HMEV (Fig. 1D).

Isolated sEV from the pasteurized pooled milk sample does not differ from HMEV of individual samples; specifically, pool HMEV express the average protein and nucleic acid levels from early and mature HMEV (Supplementary Fig. S2 and Fig. S1). We observed significant compositional heterogeneity between individual donors. Therefore, to mitigate this individual variability and to consistently model the community milk exposure that preterm infants receive in clinical donor milk bank settings, the pasteurized Pool HMEV was utilized for subsequent experiments.

### Differential intracellular accumulation of HMEV

**Fig. 2:**
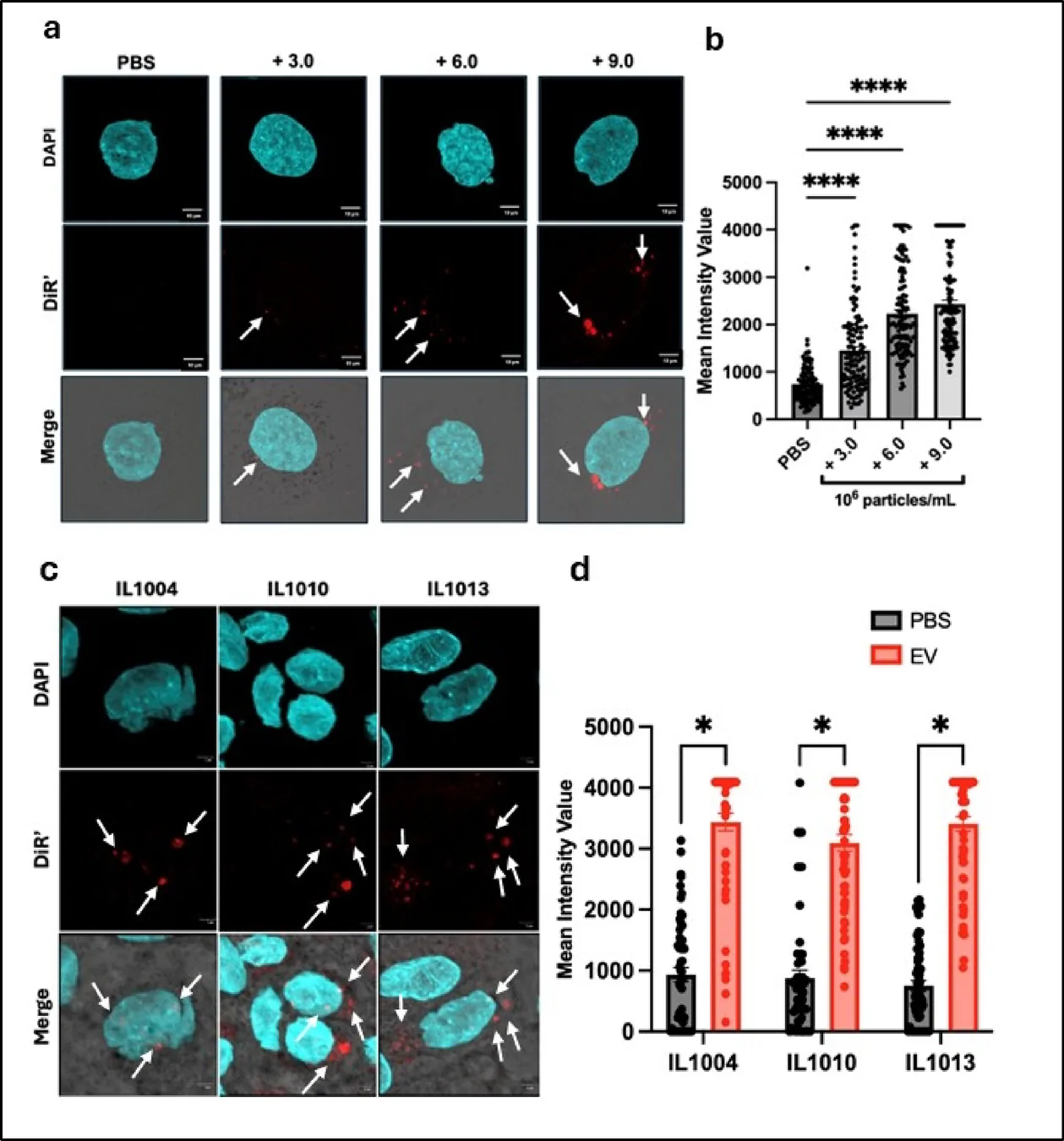
Intracellular Accumulation of HMEV in Intestinal Epithelia. HMEV were fluorescently labeled with DiIC18(7) (1,1’-Dioctadecyl-3,3,3’,3’-Tetramethylindotricarbocyanine Iodide) or DiR’ (indicated by white arrows) prior to incubation with indicated human epithelial cells grown on coverslips. (**A**) Caco-2 cells were treated with 3.0, 6.0, or 9.0 × 10^6^ pool HMEV particles and fixed at 16 h. White scale bars represent 10 μm. (**B**) FRET analysis of HMEV uptake was determined by individual ROIs from two independent cells per condition; mean intensity value were graphed with dots representing individual values on the graph. ANOVA and Tukey’s post-hoc test was conducted to identify statistical significance between the HMEV concentrations with **p-value < 0.0001. (**C**) HIE (IL1004, IL1010, IL1013) were treated with 6.0 × 10^6^ pool HMEV particles and fixed at 16 h. White scale bar represents 2 μm. (**D**) FRET analysis of HMEV uptake was determined by individual ROIs from two independent cells per condition; mean intensity value were graphed with dots representing individual values on the graph. ANOVA and Tukey’s post-hoc test was conducted to identify statistical significance between the HMEV concentrations with *p-value < 0.05.

To evaluate the intracellular kinetics of HMEV, non-cancerous mammary epithelial MCF10-2A cells were cultured in growth media with DiR’-labeled HMEV-pool for varying times (4, 8-, 16-, and 24-hours Supplementary Fig. S3A-B). Fixed cells were subject to fluorescence intensity analysis with 30–35 ROI per cell (Supplementary Fig. S4); specifically we noted a gradual increase in fluorescent-tagged HMEV with a peak in HMEV levels at 16-hours and a modest decline at 24-hours in MCF10-2A cells (Supplementary Fig. S3A and S3B), suggesting a potential timeline for HMEV initial uptake (4 h) and accumulation (16 h) in intact cells. Further, we tested how intracellular accumulation of the Pool HMEV compares to HMEV isolated from two randomly selected mature HM samples, HMEV4 and HMEV7. At 16 h in MCF10-2A cells, significantly less HMEV7 and higher HMEV4 levels accumulate compared to HMEV-pool (*p* < 0.001, ANOVA and Tukey’s post hoc test, Supplementary Fig. S5A and S5B). In fact, few intracellular HMEV7 persist at 16-hours with low fluorescence signal, similar to the background signal in PBS-Control treatment (Supplementary Fig. S5B); the low HMEV7 levels could be due to either less HMEV7 uptake or greater intracellular HMEV7 clearance than HMEV4 or Pool HMEV. Analogous to MCF10-2A cells, intracellular HMEV-pool enrichment was also noted in intestinal cells including Caco-2 cells (Fig. 2A-B). Using a dose-response treatment, we see enhanced fluorescence uptake in Caco-2 cells with increasing concentrations of labeled Pool HMEVs (Fig. 2B). We next tested HMEV uptake in human infant intestinal epithelial cells (HIE), specifically three HIEs (IL1004, IL1010, IL1013) established from ileal tissues of neonates who underwent surgery for intestinal atresia or NEC (Table 2). Five-day differentiated HIE lines were treated with either pooled HMEVs or PBS control for 24-hours. Analogous to MCF10-2A and Caco-2 cells, intracellular HMEV enrichment was observed in all infant HIEs (Fig. 2C and D). Collectively, these results support a time-dependent and dose-dependent uptake of HMEV inside cells that is variable between HMEV from different donors.

### HMEV direct inflammatory signaling signatures in infant HIEs

**Fig. 3:**
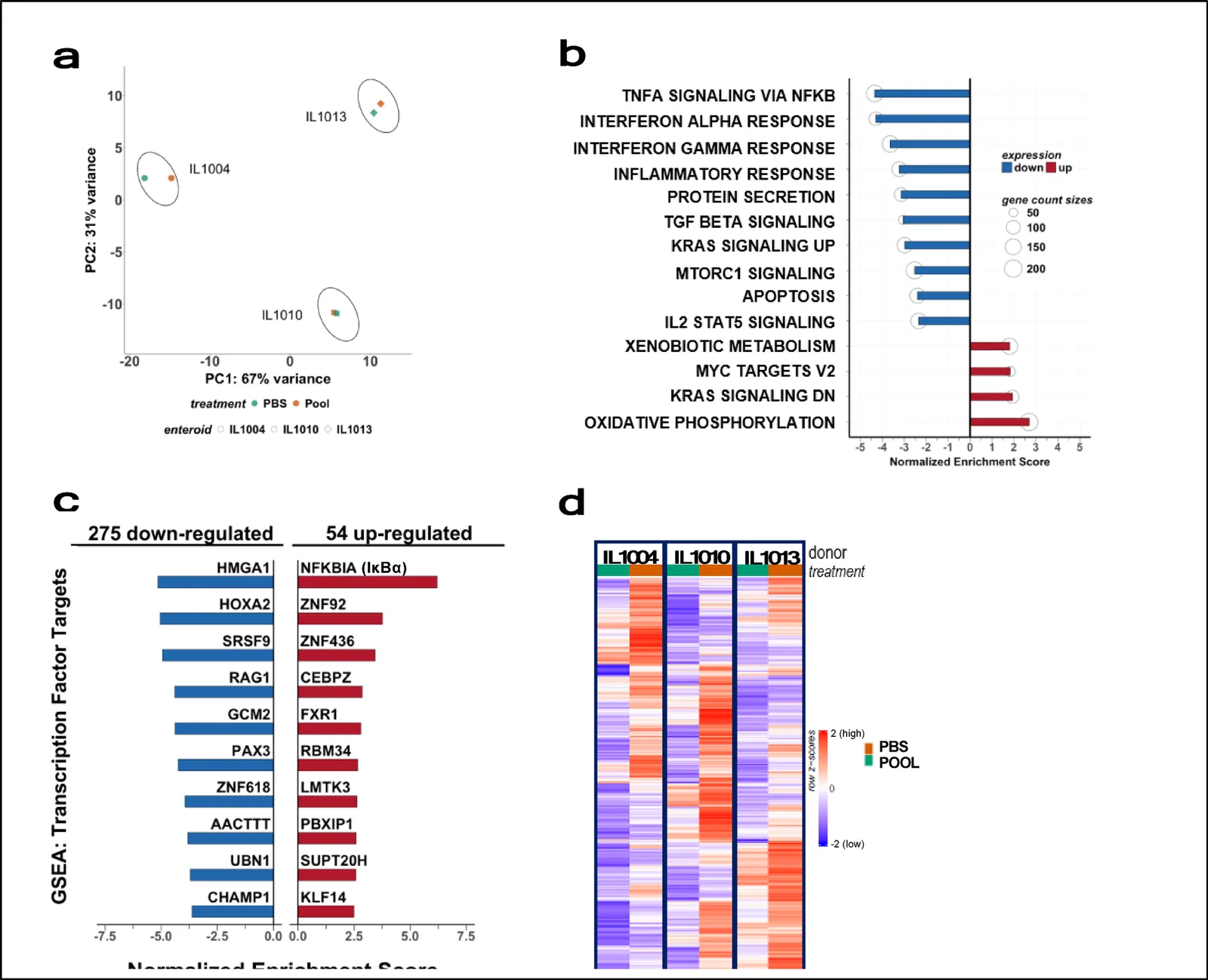
Transcriptional Response of Human Intestinal Enteroids to HMEVs. (**A**) Principal. Component Analysis (PCA) plot from RNA sequencing analysis of HIEs (IL1004, IL1010, and IL1013) after 72-hour exposure to control (PBS) or HMEV (HMEV Pool). (**B**) Top ten significantly downregulated (blue) and all significantly upregulated (red) hallmark pathways in enteroids exposed to HMEV Pool compared to the control are shown. Circles at the end of each bar represent the number of genes contributing to the pathway. (**C**) GSEA transcription factor target analysis. Normalized enrichment scores for top 10 downregulated (blue) and upregulated (red) transcription factor targets are shown. (**D**) Heat map of NFkBIA target genes between PBS and Pool treatment on the three infant HIE lines.

We next wanted to evaluate how intracellular HMEVs modulate HIE. Towards this goal, we conducted RNA-seq and bioinformatic analysis of HMEV versus PBS-control treated HIEs. Principal component analysis (PCA) showed clustering by HIE line, highlighting donor-specific differences as the major driver of transcriptional variance (Fig. 3A). While only two genes (HSPA6 and G0S2) were significantly altered (fold-change > 1.5, FDR-adjusted *p* < 0.05) across all three HIE lines, hallmark pathway analysis revealed significantly altered expression of 27 pathways (23 downregulated, 4 upregulated) with HMEV treatment. The top ten downregulated pathways and all upregulated pathways are shown in Fig. 3B. The most striking observation was that HMEVs significantly downregulated innate inflammatory pathways. The TNFα signaling via NF-κB pathway was the most significantly impacted (NES = − 4.5), followed by interferon-α response, interferon-γ response and inflammatory response (Fig. 3B).

To identify relevant drivers of the reduced inflammatory gene expression profile, we next performed GSEA transcription factor target analysis. There were 275 transcription factor targets that were significantly downregulated and 54 that were upregulated. The top ten up- and downregulated transcription factor targets are shown in Fig. 3C. Consistent with reduced TNFα signaling via NF-κB in HMEV-treated HIEs, NFKBIA (IκBα) target genes were significantly upregulated (NES = 6.2) (Fig. 3C). NFKBIA encodes IκBα, a key inhibitor of NF-κB, and its target gene enrichment suggests activation of inhibitory feedback mechanisms for pathways where NF-κB plays a role in mediating inflammatory responses. Further, a heatmap of 517 NFKBIA target genes revealed donor-specific expression patterns following HMEV treatment (Fig. 3D).

Given the strong donor-specific expression patterns, we also performed differential expression analysis using donor-aware models which allow comparisons of HMEV-treatment to the PBS control within each donor line. Using TMM normalization with edgeR quasi-likelihood testing resulted in the identification of 19 genes with FDR < 0.05, including 5 downregulated genes with fold-change *≥* 1.5. These include interferon-stimulated genes such as OASL, CXCL11 and HERC5 which showed consistent downregulation across donor-blocked analyses. A heatmap of select inflammatory and interferon-stimulated genes showed consistent downregulation across all three donor for most genes (Supplementary Fig. S6A), although the magnitude of response varied between HIE lines. Importantly, GSEA analysis remained highly consistent across both models (Supplementary Figs. S6B-S6C) with highly similar normalized enrichment scores (pairwise correlations > 0.9) and NFKBIA target genes most significantly upregulated.

**Fig. 4:**
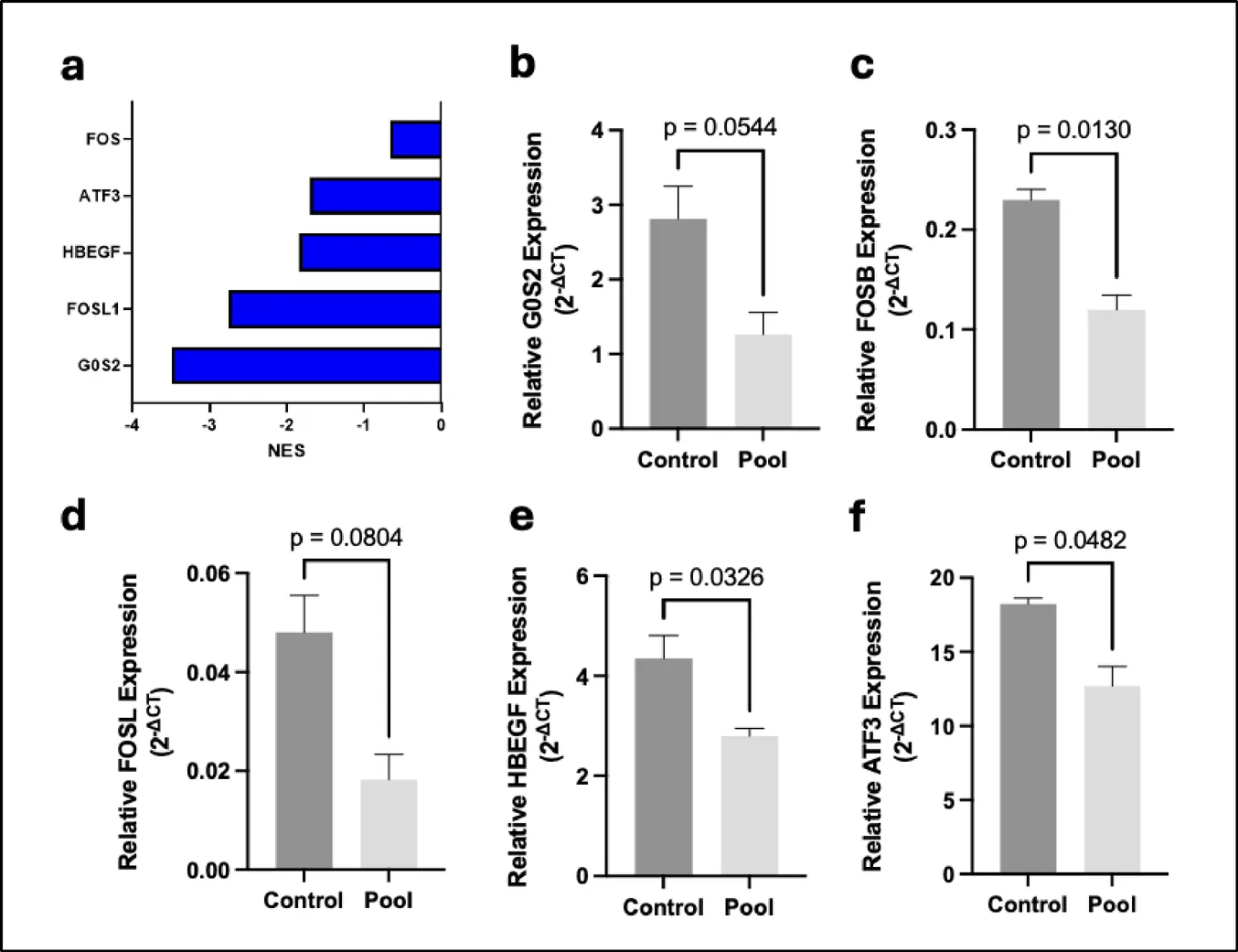
Expression of immune response genes in human intestinal enteroids exposed to HMEVs. (**A**) Normalized enrichment scores for the indicated genes. (**B**-**F**) Reverse transcription qPCR was performed on enteroids (IL1004, IL1010, and IL1013) exposed to HMEV Pool using primers for G0S2, FOSB, FOSL1, GEM, HBEGF, and ATF3. Data is represented as relative gene expression using the ΔΔCT method. Statistical significance was determined using student’s t-test (*p-value < 0.05, **p-value < 0.01).

**Fig. 5:**
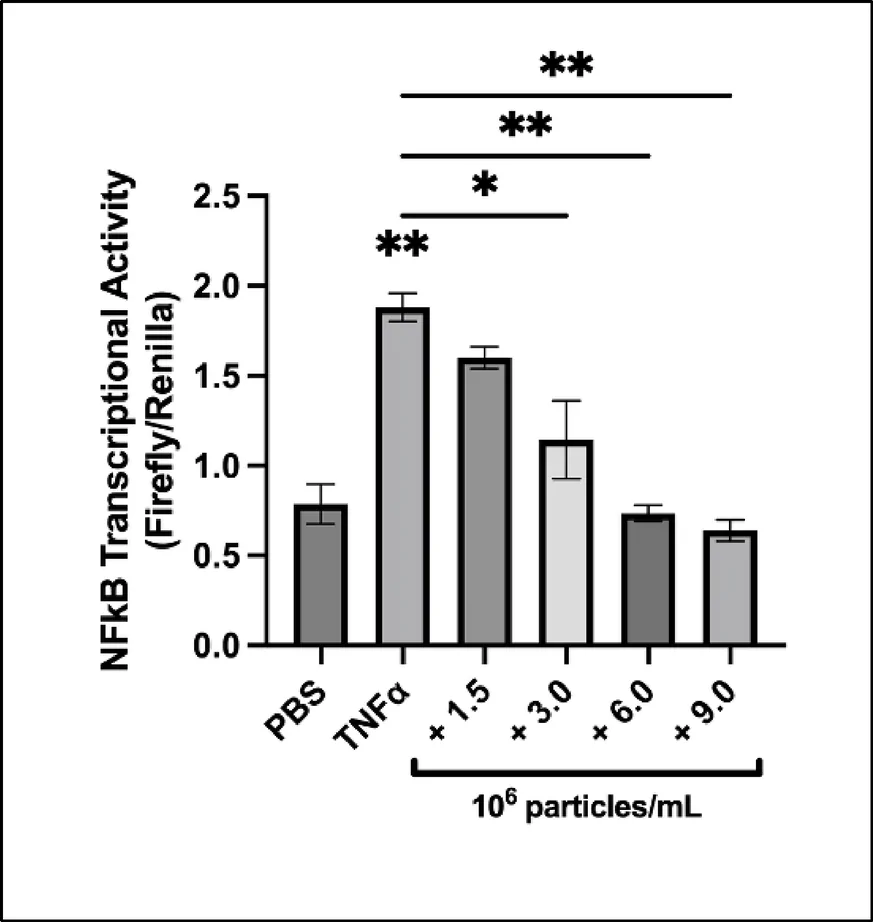
HMEV direct TNFα-induced NFκB activity. Caco-2 cells were stimulated with TNFa (25ng/mL) and subsequently treated with 1.5, 3.0, 6.0, or 9.0 × 10^6^ pool HMEV particles. Firefly and Renilla luminescence for each condition was recorded and statistical significance was reported using ANOVA and Tukey’s post hoc test (*p-value < 0.05, **p-value < 0.01).

Together, these results suggest reduced interferon and TNFα/NF-κB inflammatory signaling in HMEV-treated HIEs. NF-κB–responsive genes identified in RNA sequencing data (Fig. 4A) were further validated by quantitative PCR, which confirmed HMEV-mediated downregulation of select TNFα/NF-κB–associated transcripts, including ATF3, FOSB, FOSL1, G0S2, and HBEGF (Fig. 4B–F). To directly assess the impact of HMEV treatment on NF-κB transcriptional activity, a dual-luciferase assay was performed in Caco-2 cells. After stimulating these cells with TNF⍺ and subsequently treating them with pooled HMEVs, a dose-dependent inhibition of NF-κB signaling was observed (Fig. 5), confirming that HMEV uptake not only alters gene expression but also directly modulates inflammatory signaling pathways.

Collectively, these results demonstrate that HMEV treatment triggers an anti-inflammatory transcriptional changes in infant HIEs, characterized by the suppression of TNFα/NF-κB signaling and its downstream targets. This effect is further supported by functional assays showing direct inhibition of NF-κB activity, highlighting HMEVs as potent modulators of intestinal epithelial immune responses.

## Effects of HMEVs on epithelial barrier disruption

A hallmark of intestinal inflammation is the disruption of the epithelial barrier^24^. A previous study in Caco-2 cells demonstrated that TNFα-induced increase in permeability was mediated by NF-κB activation through downregulation of tight junction proteins and alteration in junctional localization^25^. Considering that exposure to HMEVs significantly down-regulated innate inflammatory pathways including TNFα signaling via NF-kB, we hypothesized that HMEVs could have functional consequences when the infant intestinal epithelial barrier is disrupted. To test this hypothesis, we assessed the effect of HMEV treatment on epithelial barrier disruption in infant HIEs mediated by ethylene glycol-bis(beta-aminoethyl ether)-N, N,N′,N′-tetra acetic acid (EGTA) and tumor necrosis factor-α (TNFα). EGTA is a calcium chelator that has been shown to alter paracellular permeability^26,27^. TNFα also induces an increase in intestinal epithelial permeability through NF-kB activation^25^. Consequently, the transepithelial electrical resistance (TEER) assay was used to measure paracellular ionic permeability^24^.

**Fig. 6:**
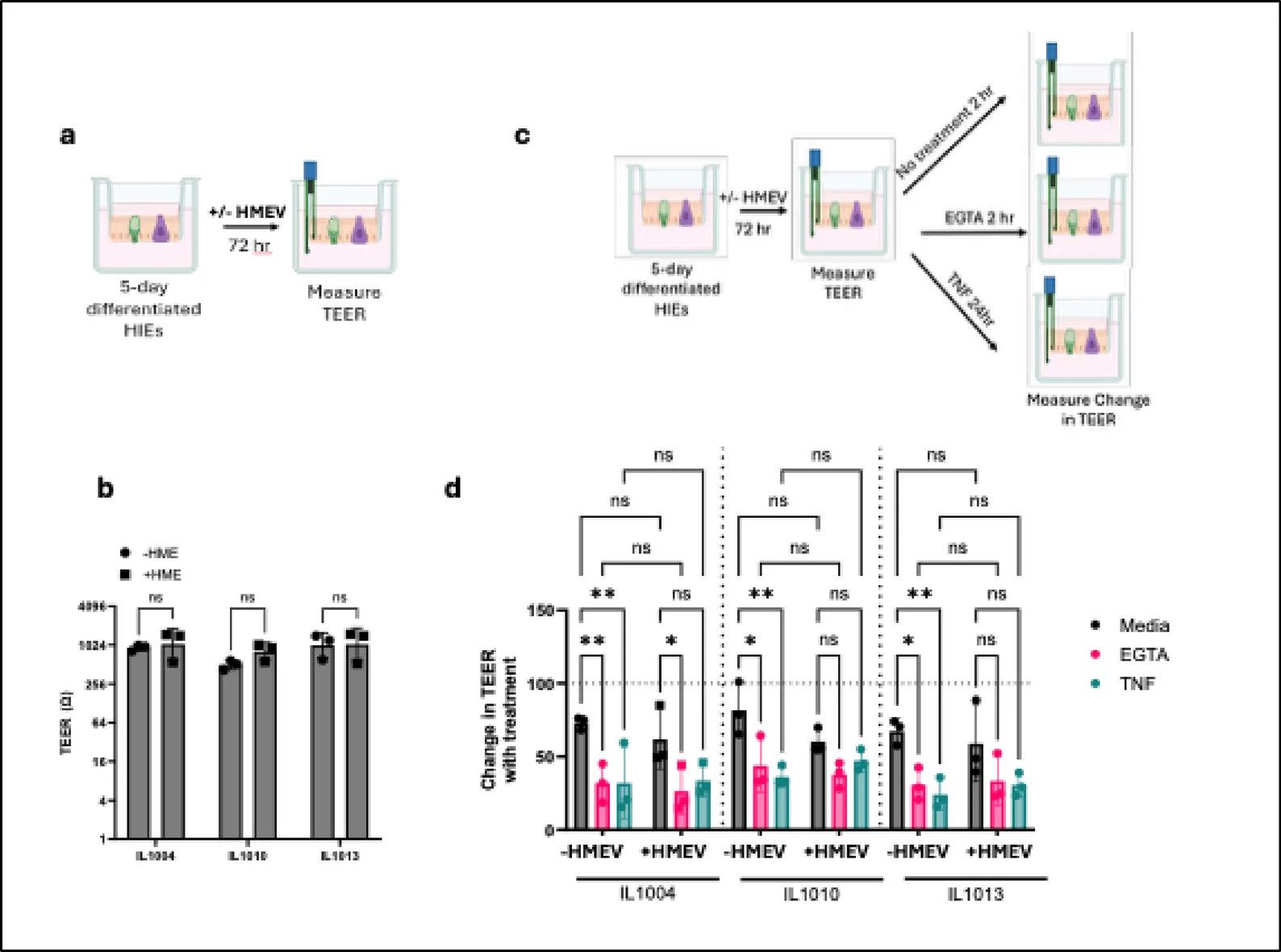
Barrier Integrity of Human Intestinal Enteroids Exposed to HMEVs. (**A**). Schematic illustrating that HIEs (IL1004, IL1010, and IL1013) were treated without and with HMEV Pool HMEV for 72-hours and TEER measured. (**B**) TEER measurements of indicated HIE in the presence or absence of HMEV; ns indicates not significant as determined with student’s t-test. (**C**) Schematic illustrating time-line of studies with barrier disruptors. HIE were either untreated or treated with Pool-HMEV for 72-hours. The HIE were then incubated in media with and without barrier disruptors EGTA or TNF. (**D**) TEER values following 2-hour exposure to media, EGTA, or TNFα are shown relative to the baseline TEER measured immediately prior to treatment (100%, dotted line). Statistical comparisons were performed among treatment conditions within each HIE line and HMEV treatment group. Two-way ANOVA with Sidak’s post-hoc statistical analysis was performed. (*p-value < 0.05, **p-value < 0.005).

The TEER values for all ileal HIEs were around 1,000 Ohms, with no significant differences in TEER were observed between HMEV treatment or no treatment for 72-hours (Fig. 6A and B). The barrier was then disrupted in the HIEs with either EGTA or TNFα with media treatment as control (Fig. 6C). A reduction in TEER was observed following incubation with control media, EGTA and TNFα for 2 h in HMEV treated and untreated HIEs (Fig. 6D). In untreated cultures, EGTA and TNFα exposure resulted in significant reductions in TEER relative to media controls across all three HIE lines. By contrast, these additional reductions were not observed in HMEV-treated cultures, with the exception of IL1004 following EGTA treatment (Fig. 6D). While direct comparisons between HMEV-treated and untreated cultures under the same challenge conditions were not statistically significant, these findings suggest that HMEV exposure may influence epithelial responses to barrier-disrupting stimuli, although the magnitude and consistency of this effect varied among donors.

## Discussion

This study demonstrates that human milk small extracellular vesicles (HMEVs) are actively internalized by infant human intestinal enteroids (HIEs) and modulate inflammatory signaling pathways. While previous studies have highlighted the anti-inflammatory potential of milk-derived vesicles, our work utilizes a physiologically relevant human intestinal organoid model to characterize their effects in the context of the neonatal gut.

### HMEV heterogeneity and rationale for pooled HMEV

Numerous studies report the heterogeneity in sEV numbers and biomolecule content isolated from human breast milk^28–31^. Characterization of HMEVs from early and mature milk revealed significant donor-specific variability in vesicle concentration and RNA content (Supplementary Fig. S1−2). Pasteurized human donor milk is utilized when raw milk from the mother is insufficient or unavailable^32^. To mitigate inter- and intra-individual heterogeneity in human milk, donor milk is pooled from a single donor or 3–7 donors at various lactation stages^33^. Consistently, we utilized pooled, pasteurized HMEVs for functional assays to model the standardized “community” milk exposure typically provided in clinical donor milk bank settings. While this pooled approach allowed us to identify core biological responses that persist across donor lines, utilizing a pooled matrix inherently risks obscuring biologically relevant, individual differences unique to specific mother-infant dyads.

### HMEV uptake

The sEV from pasteurized pooled HM are taken up and accumulate in multiple human cells (Fig. 2, Supplementary Fig. S3-5). The carbocyanine-based lipophilic fluorophore, DiR’ served as tracer in the current studies as it more efficiently intercalates into lipid sEV membrane and exhibits minimal background fluorescence, autofluorescence, and stain transfer compared to other labeling tracers^34^. Labeling of sEV coupled with fluorescent confocal microscopy presented quantifiable kinetic data that support detectable labeled HMEV in unlabeled acceptor mammary cells at 4-hours with saturation at 16-hours. These kinetics align with established timelines for human donor milk vesicle cargo tracking, where peak accumulation occurs within 5.5 to 7.5-hours in recipient cells^35^. Foundational kinetic studies frequently rely on tracking homogenous cancer cell systems (such as HeLa cells)^36^; while our work aligns with these previous reports, it also notably extends these findings by evaluating heterogeneous vesicles derived directly from human milk across multiple epithelial models, including human neonatal HIEs.

### HMEV-induced inflammatory signaling and functional changes

A key finding from our study was that HMEVs modulate transcriptional signatures associated with inflammation in the neonatal intestinal epithelium (Figs. 3 and 4, Supplementary Fig. S6). Previous studies using mouse intestinal organoid and in vivo rodent models consistently demonstrate that HMEV mitigate hypoxia and LPS-induced intestinal inflammation by downregulating key pro-inflammatory genes like IL-6, TNFa, and TLR4^6,10^. The HIEs used for our study were established from human neonates presenting with NEC and intestinal atresia and inflammatory markers are known to be elevated in both disease states^37,38^. In particular, NF-κB-mediated signaling and secretion of pro-inflammatory cytokines such as TNFα are elevated in NEC. Thus, our findings of HMEV-driven significant downregulation of inflammatory pathways in neonatal HIEs is significant and presents a valuable platform for assessing HMEV-based interventions.

Our transcriptomic analysis demonstrates HMEVs exert a broad anti-inflammatory effect on the human infant intestinal epitheliums with pathway-level analyses exhibiting suppression of innate immune and inflammatory programs. Hallmark gene set enrichment analysis highlighted downregulation of *TNFα signaling via NF-κB* as the most significantly affected pathway, along with reduced expression of interferon-α, interferon-γ, and overall inflammatory response pathways. These data suggest that HMEVs attenuate major inflammatory cascades in neonatal intestinal epithelia. Additional insight was obtained from the transcription factor targets where we observed significant upregulation of *NFKBIA*(IκBα) target genes, consistent with enhanced inhibitory feedback on NF-κB signaling^39,40^. Hence, enrichment of IκBα-regulated programs observed in the current study provides support to the hypothesis that NF-κB activity is restrained in HMEV-treated HIEs. This interpretation is further validated by RT-qPCR analysis showing reduced transcript abundance for several canonical NF-κB–responsive genes, including *ATF3*,* FOSB*,* FOSL1*,* G0S2*, and *HBEGF.* Additionally, dual-luciferase assays with dose-dependent inhibition of NF-κB signaling was observed in HMEV-treated Caco-2 cells. This dual trend of diminished primary inflammatory cascades and elevated feedback transcripts suggests a mechanism by which HMEVs may modulate epithelial responses to promote mucosal homeostasis.

Additionally, we examined whether HMEVs influence epithelial responses to experimentally induced barrier disruption (Fig. 6). Previous studies have demonstrated that pro-inflammatory signaling can impair epithelial barrier integrity through disruption of tight junctions^41–43^. We previously used EGTA and TNFα to compare differences in barrier function between infant and adult jejunal HIEs and demonstrated that infant HIEs may be more leaky following barrier disruptions. EGTA treatment has also been associated with increases in mRNA transcripts of TNFα and other proinflammatory cytokines^44^. In the present study, HMEV exposure did not alter baseline TEER measurements in ileal HIEs. Following challenge with EGTA or TNFα, significant reductions in TEER were observed in untreated cultures, whereas these reductions were less consistently observed in HMEV-treated cultures. While direct differences between HMEV-treated and untreated cultures were not significant, our findings suggest that HMEVs may modulate epithelial responses to barrier-disrupting stimuli. These observations are consistent with prior reports suggesting beneficial effects of milk-derived extracellular vesicles on intestinal epithelial barrier in transformed cell lines and animal models function and support further investigation in physiologically relevant infant intestinal models.

## Limitations and Future Directions

Several limitations of this work must be noted. First, the specific bioactive cargo (proteins, RNAs, or lipids) responsible for the observed transcriptional changes remains to be identified. Furthermore, although lactation-stage variation of biomolecules (including CD63 and C9 protein in Fig. 1) was observed, the precise regulatory control mechanisms governing active protein and RNA packaging into HMEVs remain poorly defined. Second, pooling donor milk reduces variability but may obscure biologically relevant heterogeneity among individual mother–infant dyads. Future translational studies must balance the baseline reproducibility of pooled clinical matrices with targeted, single donor lines to fully map how maternal phenotypes dictate infant gut maturation. Third, we observe heterogeneity in HMEV responses between HIE donors. This is likely reflective of real-world differences in infant responses to treatments and suggests the need for inclusion of additional HIE lines in future studies. While our data establishes a strong association between HMEV exposure and reduced inflammatory signatures, further “omics” studies are required to isolate the active components. Additionally, while HIEs provide a robust model of the human epithelium, they lack the complex immune and microbial interactions present in vivo.

In conclusion, our results provide a technically sound framework for understanding how HMEVs influence neonatal intestinal homeostasis. By dampening NF-κB-mediated inflammation and supporting barrier integrity in a donor-dependent manner, HMEVs likely contribute to the protective benefits of human milk in the developing gut.

## Methods

### Human milk samples

All methods involving human participants and human samples were carried out with relevant guidelines and regulations. All experimental protocols were reviewed and approved by the University of Houston Institutional Review Board (IRB protocol # STUDY00004613) and informed consent was obtained from participants. Eight HM samples were collected from deidentified donors at Texas Children’s Hospital or healthy lactating volunteers (Table 1). The individual milk samples were categorized according to the United States Department of Agriculture (USDA) stages of milk (Table 1); specifically, 3 samples (HMEV1, HMEV2, and HMEV3) were identified as early (colostrum/transitional) milk with collection from birth up to 21 days postpartum. In contrast, samples HMEV4, HMEV5, HMEV6, HMEV7, and HMEV8 collected greater than 22 days postpartum are identified as mature HM samples.

**Table 1:**
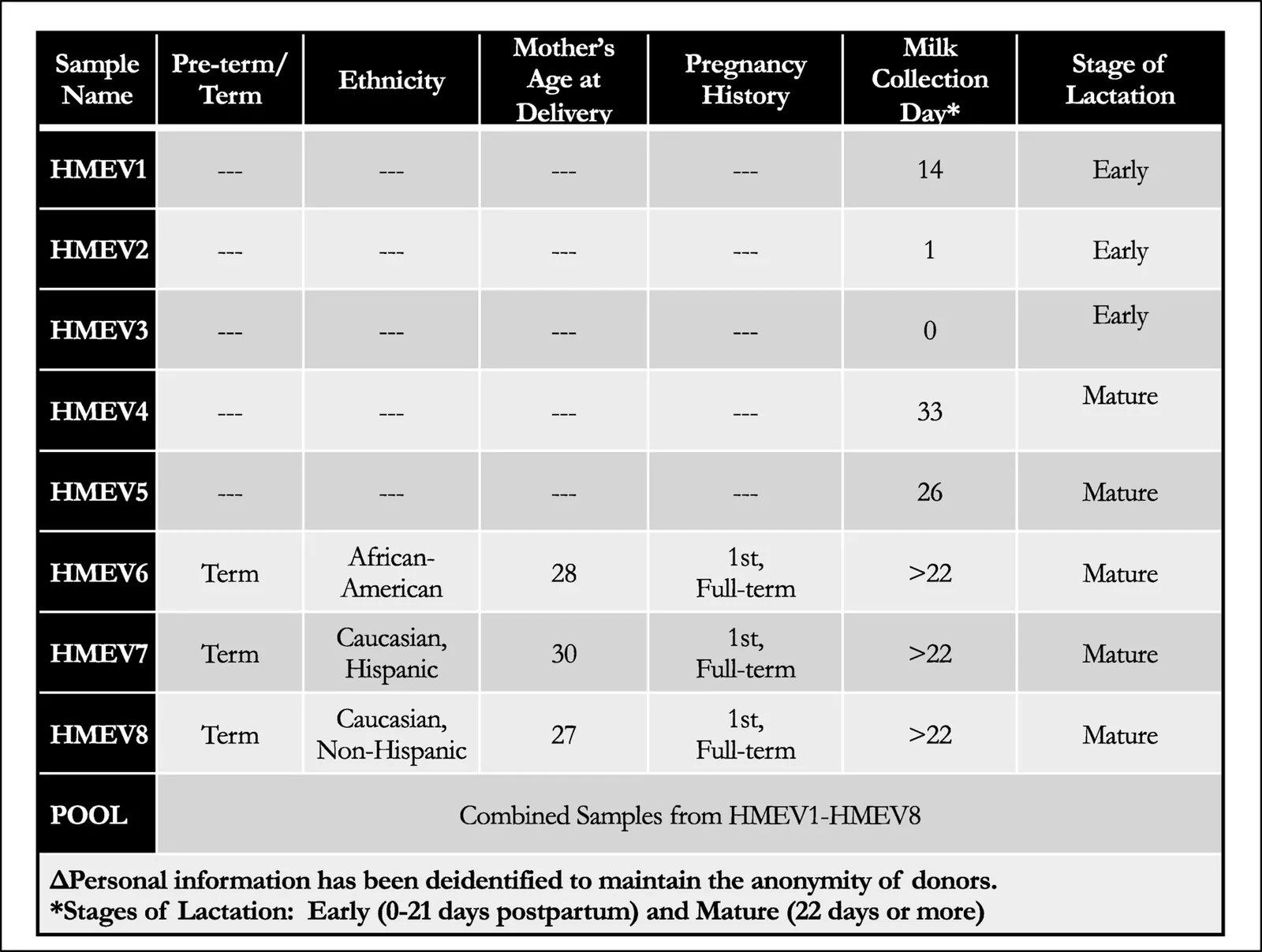
Human milk samples.

Individual samples were aliquoted upon receipt in the laboratory and stored at −80˚C to avoid multiple freeze-thaw cycles. Additionally, the eight individual HM samples were combined and processed analogous to samples at HM donor banks as outlined by the Human Milk Banking Association of North America (HMBANA)^45^. Briefly, equal volumes of individual milk were combined and homogenized to ensure efficient distribution. Homogenized HM samples were then pasteurized with incubation at 65˚C for 30 min and immediately transferred to an ice bath. This combined, pasteurized (referred to as Pool) HM sample was then stored at −80˚C.

### Human milk-derived sEV isolation

Milk samples were clarified through sequential centrifugation cycles. The sEV were isolated from clarified milk as previously described^46^. Isolated sEV were immediately used for downstream analysis or stored at −80˚C until subsequent use.

### Cell lines and human intestinal enteroids

MCF10-2 A cells were purchase from ATCC and cultured in DMEM: F12 media supplemented with insulin, EGF, cholera toxin, hydrocortisone, 5% horse serum and 1% penicillin/streptomycin. Caco-2 cells were purchased from ATCC and cultured in DMEM: F12 media supplemented with 5% horse serum and 1% penicillin/streptomycin. Infant HIEs were established from ileal intestinal crypts isolated from surgical tissues and cultured, as previously described^20^. Demographic details and reasons for surgery for three infant HIE donors are presented in Table 2. For all studies, HIEs were plated as monolayers on transwells at a density of 2.5 × 10^5^ cells per transwell and differentiated using in-house differentiation media for 5 days. HMEVs were diluted in media appropriate for cell line, with 3–5 µg of HMEVs added in each experiment.

**Table 2:** Human infant intestinal enteroids.

Line	Ethnicity	Gestational age (weeks)	Age at surgery (weeks)	Corrected gestational age (weeks)	Gender	Surgical diagnosis
IL1004	African-American Caucasian,	23	12	35	M	Type I intestinal atresia
IL1010	Non-Hispanic	37	20	57	M	Intestinal atresia
IL1013	Caucasian, Hispanic	23	12	35	F	Necrotizing enterocolitis (NEC)

### Nucleic acids and protein quantification

DNA was isolated from HMEV lysis using phenol-chloroform organic extraction, and total RNA was extracted using the Direct-zol RNA Miniprep (Zymo Research, Irvine, CA USA). Protein concentration was determined as described before^47–49^.

### SDS-PAGE and immunoblotting

Proteins were extracted from individual and pool HMEV using lysis buffer. Isolated HMEV samples were separated on SDS-PAGE gels and then blotted to PVDF membranes (Bio-Rad, Hercules, CA, USA). Membranes were blocked and exposed to primary antibodies including the Calnexin antibody (ABclonal, Woburn, MA, USA; A4846), CD9 antibody (Santa Cruz Biotechnology, Dallas, TX, USA; sc-13118), CD63 antibody (ABclonal, Woburn, MA, USA; A19023), and HSP70 antibody (Santa Cruz Biotechnology, Dallas, TX, USA; sc-24). After incubation with an appropriate secondary antibody (mouse sc-525409, or rabbit sc-2357, Santa Cruz Biotechnology, Dallas, TX, USA), proteins were detected on membranes using the chemiluminescence substrate (SuperSignal west Pico Plus reagent, ThermoScientific, Rockford, IL, USA) and imaged. Densitometric analysis of immunoblots were conducted using ImageJ as described before^47–49^.

### Nanoparticle tracking analysis

Nanoparticle tracking analysis measurements were performed with the NanoSight LM14C (Malvern Panalytical, Malvern, WR UK) to determine the size and concentration of HMEV enrichment. The system was calibrated with 100 nm polystyrene beads (Nanosphere Size Standards, ThermoFisher Scientific, Waltham, MA, USA) and then diluted in Millipore water prior to each run. Capture settings were 1.5 screen gain and camera level 15, syringe speed 100, cell temperature maintained at 22.5 ˚C for all measurements. Each sample was diluted in a 500 to 4000-fold range to a final volume of 1 mL in 0.22 μm filtered 1X PBS. The system was washed with 1X PBS between samples.

### DiR’-labeling of HMEV and cellular uptake

Isolated HMEV were labeled using lipophilic carbocyanine tracer, DiIC18(7) (1,1’-Dioctadecyl-3,3,3’,3’-Tetramethylindotricarbocyanine Iodide) or DiR’(Invitrogen). The appropriate volume of DiR’ stock was introduced to PBS diluted HMEV to reach a 2µM final concentration of the tracer. Samples were incubated in the dark for 15 min. Following incubation, DiR’-labeled HMEV were dispersed using a pipette to reduce the formation of aggregates. Labeled HMEV were introduced to media appropriate for each cell line and incubated for at least 20 min at 37˚C. Post incubation, cultured cells were washed with cold PBS and fixed to coverslips using 4% paraformaldehyde in PBS solution. After washing and fixation, cells were mounted to glass microscope slides using mounting medium with DAPI. Imaging of fixed cells was performed using confocal microscopy (Olympus FV3000). For analysis, fluorescence intensity was quantified by identifying 30–35 ROIs per cell. Fluorescence intensity was measured for at least two cells per condition. The fluorescence intensity for each ROI was obtained and the overall average mean fluorescence intensity was determined for each condition.

### NFkB dual-luciferase reporter assay

Cultured Caco2 cells were seeded into a 24-well plate and co-transfected with NFκB (IgK3) firefly luciferase plasmid and Renilla plasmid in a 1:100 ratio using Lipofectamine 2000 for 24 h. After 24 h, cells were treated with PBS, TNF⍺, and increasing particle concentrations of isolated EVs for 24 h. Following treatment incubation, cells were washed gently with PBS and were lysed using 1X Passive Lysis Buffer (PLB) with gentle rocking for 15 min at room temperature. Firefly and Renilla luciferase activities were sequentially quantified using the Dual-Luciferase^®^ Reporter (DLR™) Assay System (Promega) according to the manufacturer’s protocol. Luminescence was recorded using a Victor X4 Multilabel Plate Reader (PerkinElmer) with three technical replicates per biological well. Final NFκB activation is expressed as the Firefly/Renilla ratio relative to the vehicle (PBS) control.

### RNA sequencing and analysis

Total RNA was extracted using the RNeasy Mini Kit (Qiagen, Redwood City, CA USA) from HIE monolayers. Paired-end Illumina sequencing was performed by Novogene.

Quality checks on raw sequence reads were performed using FASTQC package ver. 0.11.9. Illumina adapters and low-quality basepairs were trimmed using TrimGalore ver. 0.6.5. Trimmed reads were then aligned to human genome build GRCh38.98 using HiSAT2 ver 2.2.1^9^. A count matrix was generated from the aligned reads using feature counts. Principal component analysis of feature count data was performed using DeSeq2 package^26^.The results were visualized using R package ggplot2^27^. Sequencing data files and raw count matrix files will be deposited in the Gene Expression Omnibus (see note in Data Availability). The EdgeR ver. 3.32.1 R package was used to perform differential gene expression analysis for the protein coding genes^28^. FDR-adjusted p-value < 0.05 and fold-change exceeding 1.5x was used to identify significantly differentially expressed genes. Enriched pathways were determined using Gene Set Enrichment Analysis (GSEA) v 3.3^29^using the Gene Ontology pathway compendium compiled by the MsigDB database^30^. The GSEA analysis was performed on rank files comprised of gene symbols and the corresponding log2 fold changes for all the expressed genes. Adjusted p-value FDR < 0.25 was considered significant.

### Real-time PCR

Real-time PCR studies were conducted as before^47–49^. Briefly, total RNA was extracted using PureLink RNA Mini Kit (ThermoFisher Scientific, Waltham, MA, USA) and converted to cDNA with an iScript cDNA Synthesis Kit (Bio-Rad, Hercules, CA, USA) according to the manufacturer’s instructions. Primer sequences are listed in Supplementary Table 1 (IDT, Coralville, IA, USA). RT-PCR was performed using the SsoAdvanced Universal SYBR^®^ Green Supermix (Bio-Rad, Hercules, CA, USA) and 7500 Fast Real-Time PCR System (Applied Biosystems, Waltham, MA, USA). Data were normalized to endogenous controls, GAPDH and 18 S, and analyzed using either ΔCT or ΔΔCT methods.

### Barrier integrity assessment

HIEs were plated as monolayers on transwells and differentiated for 5 days as described previously^20^. Transepithelial electrical resistance (TEER) was measured following addition of 5 µg of HMEV or PBS for 72-hours. HIEs were then washed with PBS and either 5 mM EGTA, pH 7.4 diluted in differentiation media or differentiation media alone as control was added to apical side of the transwells. The cultures were incubated at 37 °C for 2-hours and TEER was measured as described previously^20^. Alternatively, 0.25 µg/mL of TNF-α prepared in HIE differentiation media was added to the basolateral side of the HIE transwells for a 24-hours period^20^. TEER was again measured following treatment and changes in barrier integrity was assessed.

### Statistical analysis

Significant differences were assessed on a minimum of 2 independent repeat experiments with two-tailed student’s *t-test* or one-way ANOVA as appropriate. With ANOVA, subsequent Tukey’s post-hoc test were used to determined difference between the groups. Statistical significance was considered by a *p*-value < 0.05.

## Supplementary Information

Below is the link to the electronic supplementary material.

Supplementary Material 1

## Data Availability

Sequencing data files and raw count matrix files was deposited in the Gene Expression Omnibus (GEO accession GSE318686, https:/www.ncbi.nlm.nih.gov/geo/query/acc.cgi? acc=GSE318686) and made publicly available.
