# Supplementary material for: Human milk small extracellular vesicles elicit changes in inflammatory response in infant human intestinal enteroids
Source: Sci Rep. 2026 Jun 26;16:29400. doi: 10.1038/s41598-026-59527-2 (PMC13594198; doi:10.1038/s41598-026-59527-2)
Supplement: Supplementary file 1 [file 41598_2026_59527_MOESM1_ESM.pdf]

SUPPLEMENTARY TABLE

Supplementary Table 1. qPCR Primer Sequences

Target	Primer Sequence	Reference
ATF3	Forward: 5’-TTCTGAGCCCGGACAATACAC-3’<br>Reverse: 5’-AAGAACGAGAAGCAGCATTTGAT-3’	[Xie 2021]
FOSB	Forward: 5'-TACTCCACACCAGGCATGAG-3'<br>Reverse: 5'-CTTCGTAGGGGATCTTGCAG-3	[Park 2019]
FOSL1	Forward: 5'-AGAACCCAGCAGCCGTGTA-3'<br>Reverse: 5'-ATGGTGTTGATGCTTGGCAC-3'	[Kitajima 2023]
GEM	Forward: 5'-GAACTAGGCTCATCAGAATCGTGAC-3'<br>Reverse: 5'-GAGCTGTGACATACAAGGGTCAACC-3'	[Chevalier 2014]
G0S2	Forward: 5'-AGGAGATGATGGCCCAGAAG-3’<br>Reverse: 5'-AGGGCTTGCTTCTGGAGAG-3'	[Wang 2021]
HBEGF	Forward: 5’-GTGACTTGCAAGAGGCAGAT-3’<br>Reverse: 5’-GTGCTCCTCCTTGTTTGGT-3'	[Cramer 2019]
18S	Forward: 5’-AGAAACGGCTACCACATCCA-3’<br>Reverse: 5’-CACCAGACTTGCCCTCCA-3'	

SUPPLEMENTARY FIGURES

MP4 File uploaded.

Supplementary Movie 1. Movie of DiR'-labeled HMEV dispersion in MCF10-2A cells. Independent z-stack images of representative MCF10-2A cells exposed to DiR'-labeled HMEV Pool were combined to show distribution of labeled HMEV.

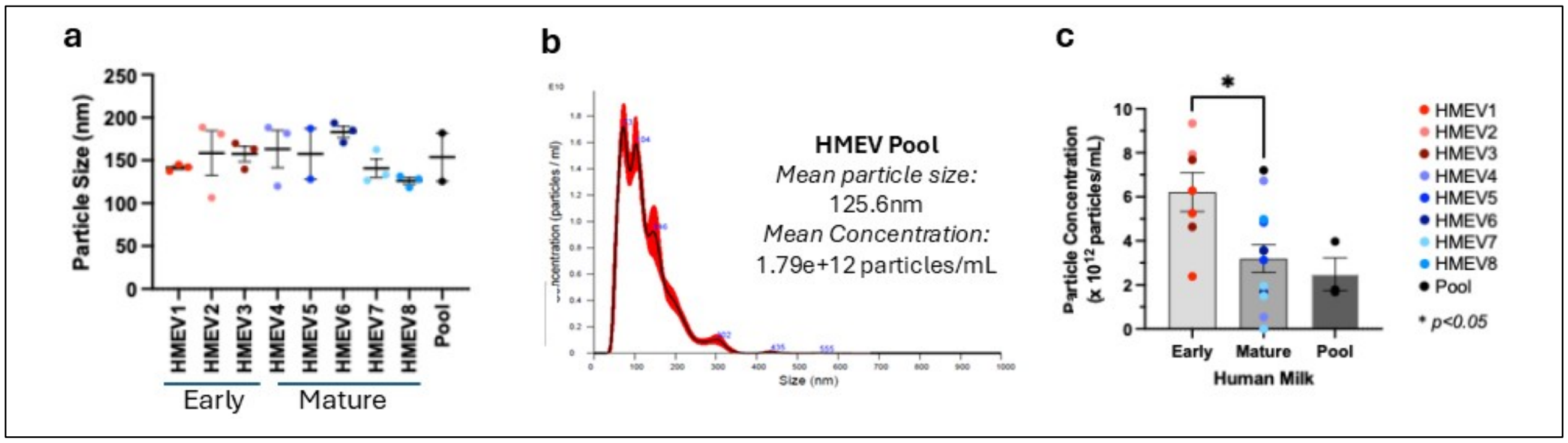

Supplementary Figure 1. Characteristics of Extracellular Vesicles Isolated from Mature, Early and Pool Human Milk. A-C. The sEV were isolated from individual raw human milk samples and the representative donor pool samples. Nanoparticle tracking analysis was performed on three separate sEV isolation preparations for each individual and pool sample. A. Mean particle size (nm) was graphed for each individual preparation and the average of all three are represented here with horizontal lines. There was no significant difference between mean particle size of each individual HMEV sample compared to the mock donor pool (ANOVA). B. Representative figure of nanoparticle tracking analysis. C. The concentration of particles (particles/mL) in HMEV from early, mature, and pool was compared using ANOVA and Tukey's post-hoc test; asterisk represents significant difference of $p<0.05$.

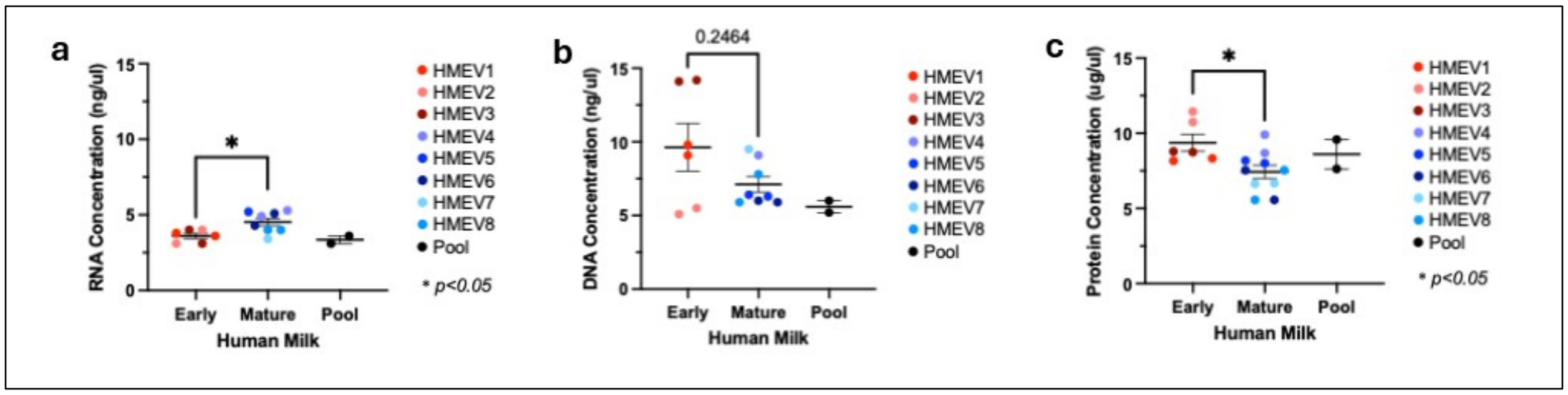

Supplementary Figure 2. Biomolecule Cargo of Mature, Early and Pool HMEVs. Average (A) RNA, (B) DNA and (C) protein concentrations of two independent HMEV preparations. ANOVA and Tukey's post-hoc tests were used to test for statistical significance between HMEV isolated from early, mature, and pooled samples; the p-value is indicated over the bracket or represented with asterisks if $p<0.05$.

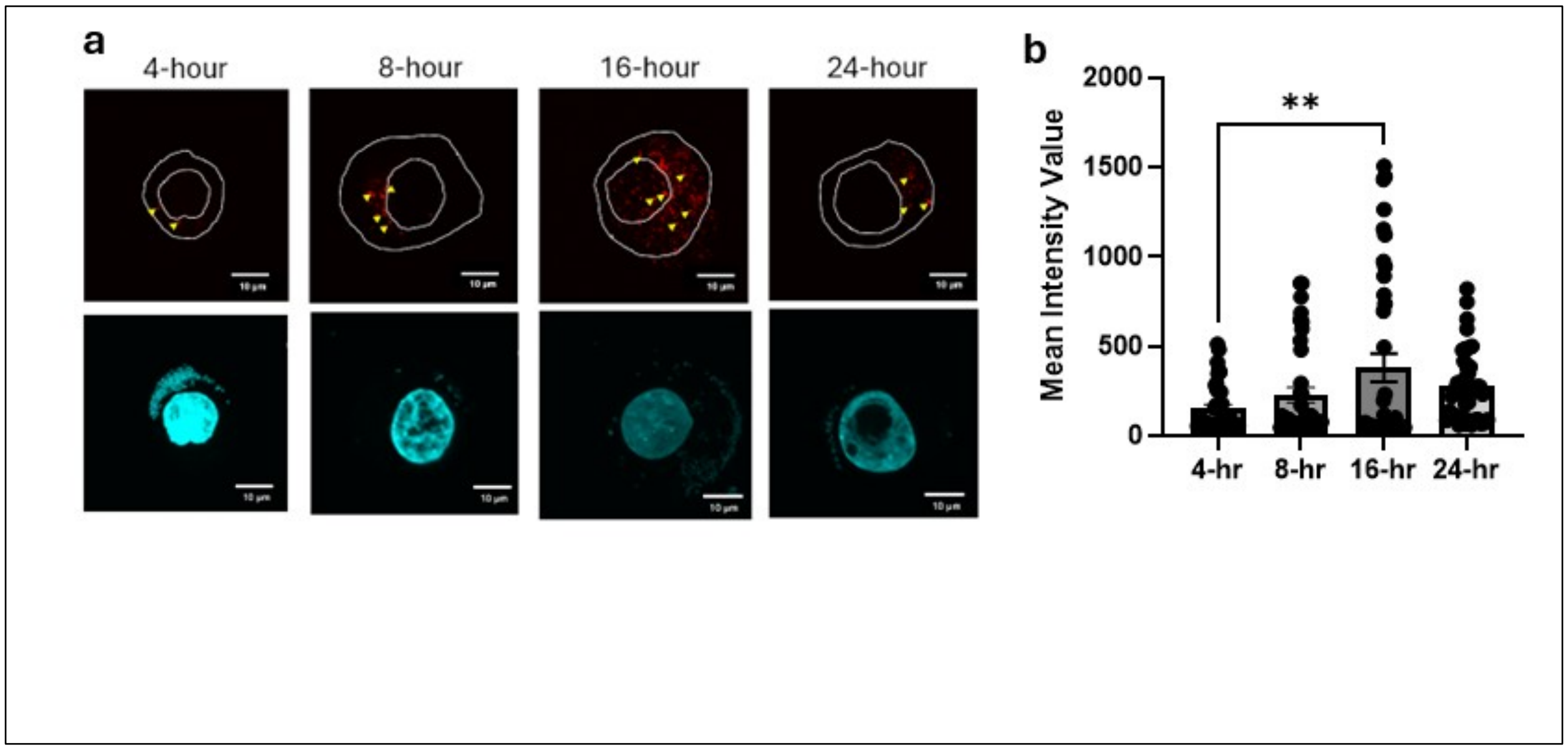

Supplementary Figure 3. Time-Dependent HMEV Uptake. HMEV were fluorescently labeled with DiIC18(7) (1,1'-Dioctadecyl-3,3,3',3'-Tetramethylindotricarbocyanine Iodide) or DiR' (indicated by yellow arrows) prior to incubation with indicated human epithelial cells grown on coverslips. (A) MCF10-2A cells were fixed at 4 hours, 8 hours, 16 hours, or 24 hours with Pool-HMEV. White scale bars indicate 10 µm. (B) FRET analysis of HMEV uptake was determined by individual ROIs from two independent cells per condition; mean intensity value were graphed with dots representing individual values on the graph. ANOVA and Tukey's post-hoc test was conducted to identify statistical significance between the time points with **p-value<0.01.

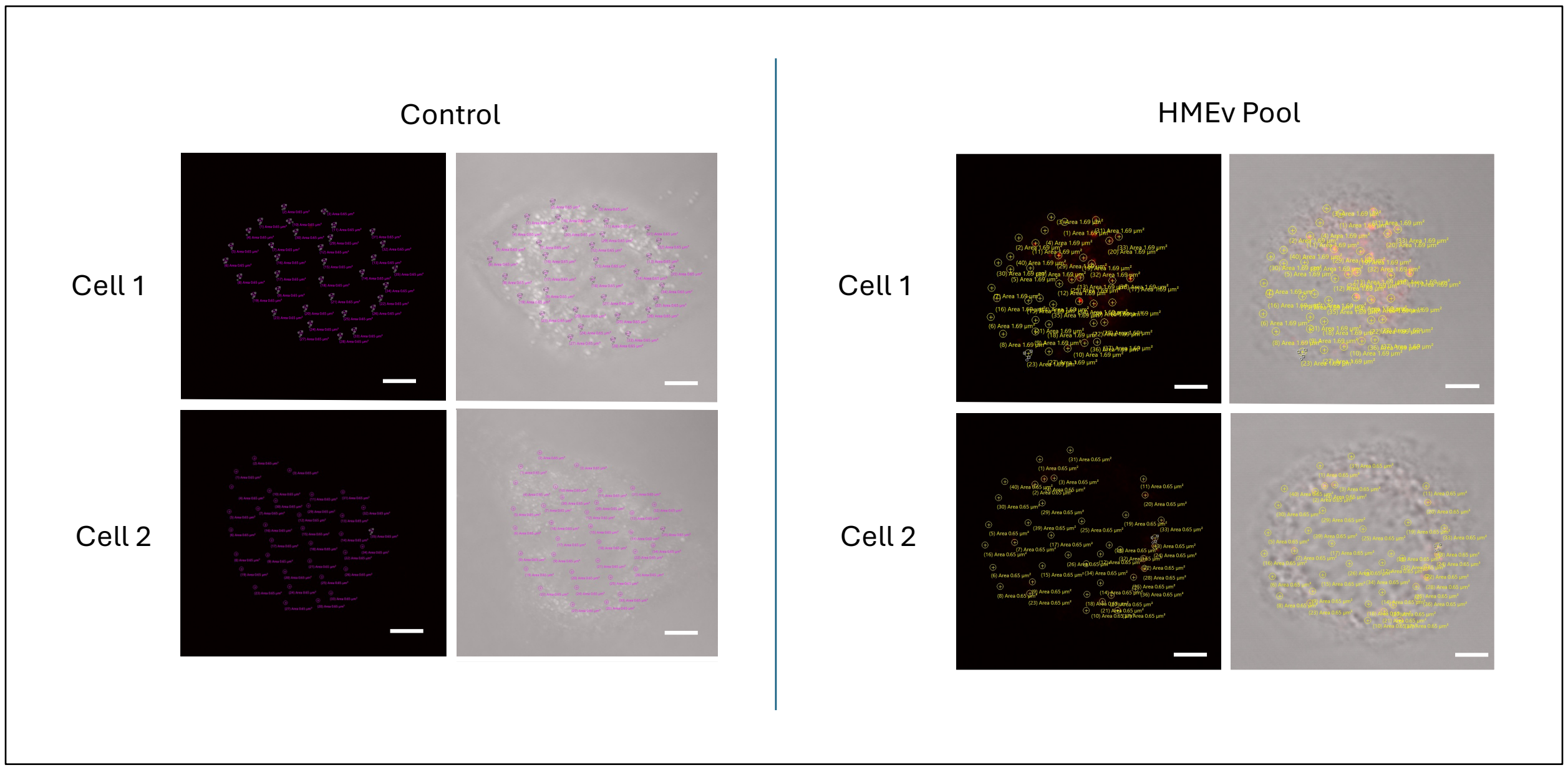

Supplementary Figure 4. ROIs to calculate florescence intensity. Thirty to thirty-five regions of interest (ROIs) with an area of 0.65 µm2 were identified for at least two cells per condition.

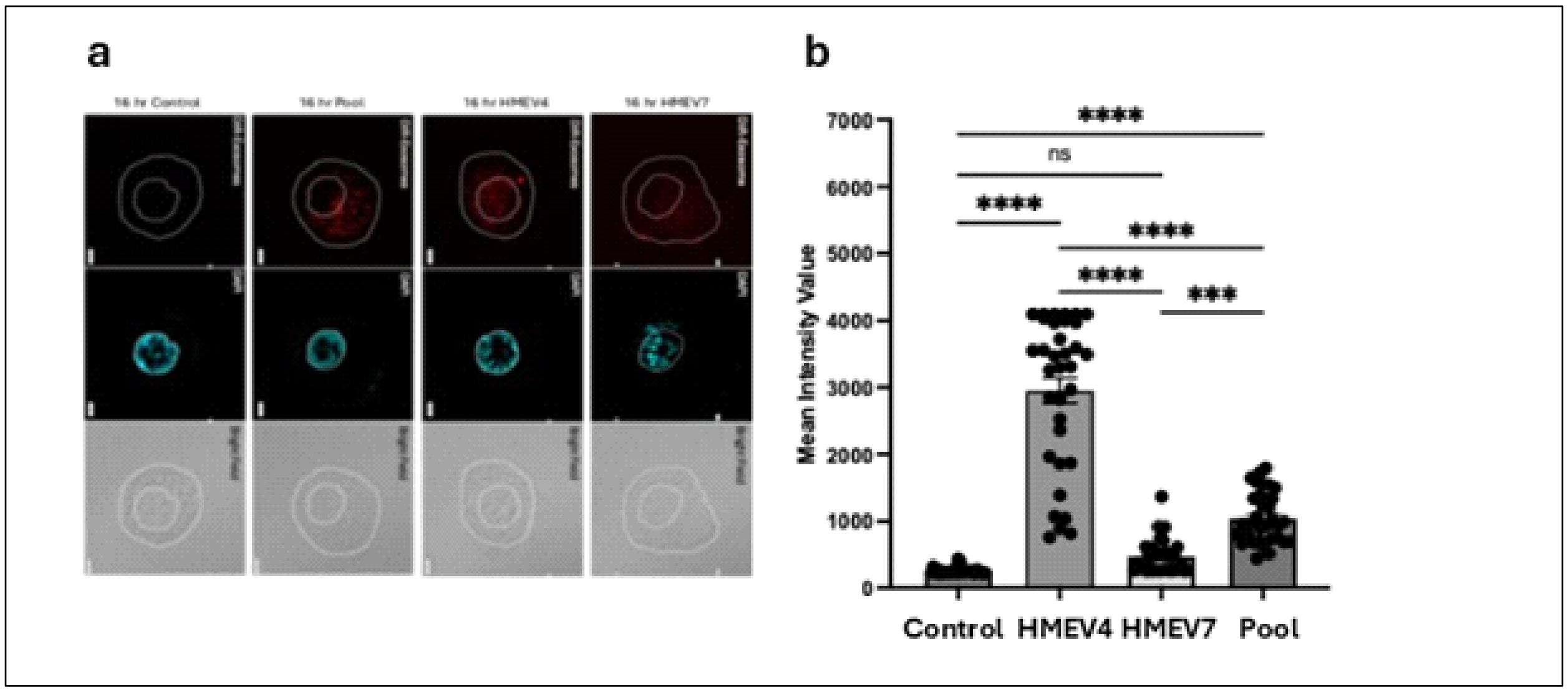

Supplementary Figure 5. Intracellular Accumulation of pool and individual-donor HMEV. (A) MCF10-2A were incubated with fluorescently labeled HMEV4, HMEV7, Pool-HMEV, or PBS-Control for 16-hours. White scale bars indicate 10 µm. (B) Mean intensity value is determined by individual ROIs from two independent cells per condition (represented by individual dots on the graph). Ordinary one-way ANOVA with Tukey's post-hoc statistical analysis was performed. (***p-value<0.001, ****p-value<0.0001, ns= not significant).

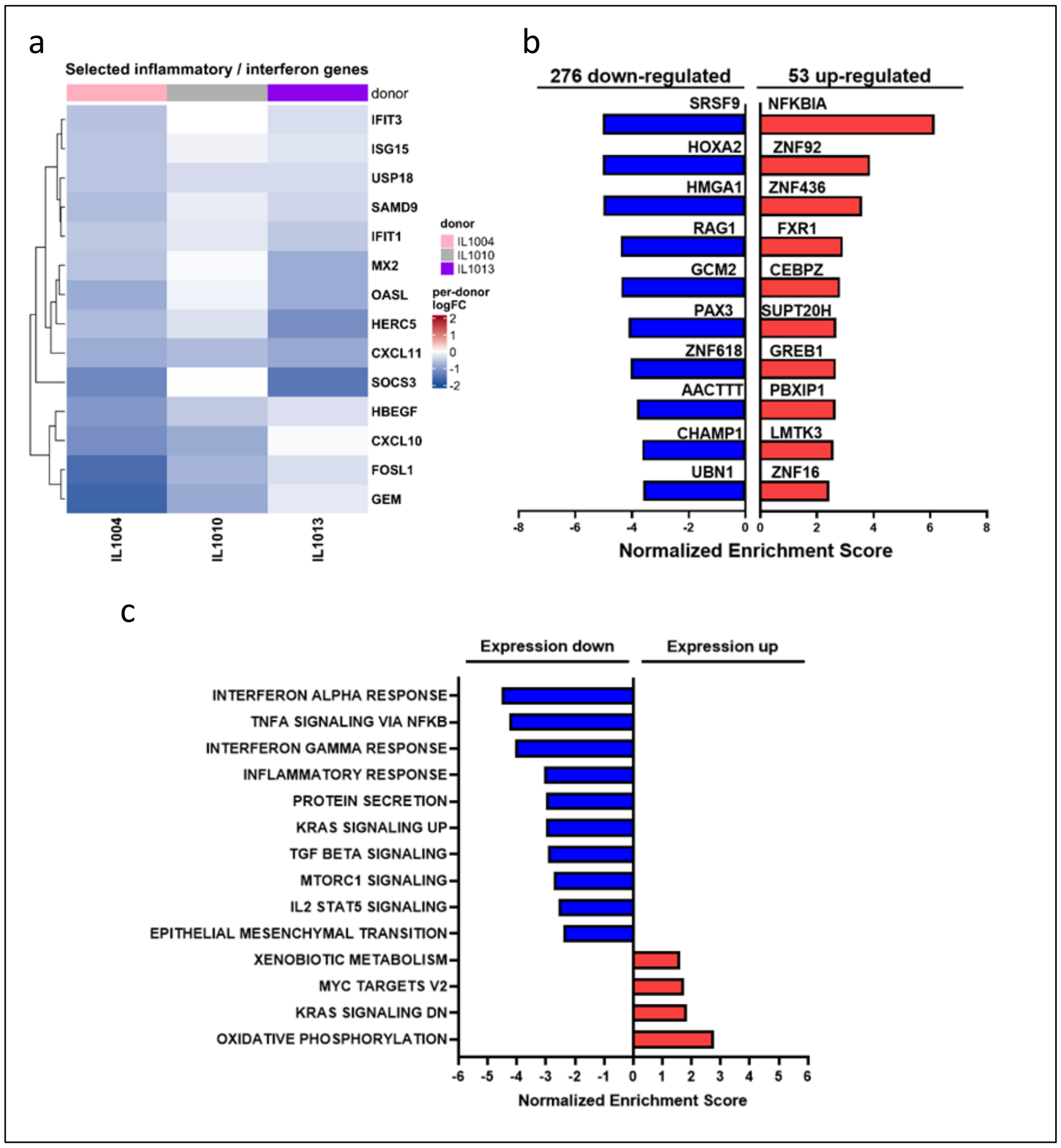

Supplementary Figure 6. Transcriptional Response of Human Intestinal Enteroids to HMEVs Using TMM normalization with edgeR quasi-likelihood testing

(A) Per-donor log fold change (HMEV Pool vs PBS) heatmap for select inflammatory and interferon-related genes across three HIE lines. (B)Top ten significantly downregulated (blue) and all significantly upregulated (red) hallmark pathways in HIEs exposed to HMEV Pool compared to the control are shown. (C) GSEA transcription factor target analysis. Normalized enrichment scores for top 10 downregulated (blue) and upregulated (red) transcription factor targets are shown.

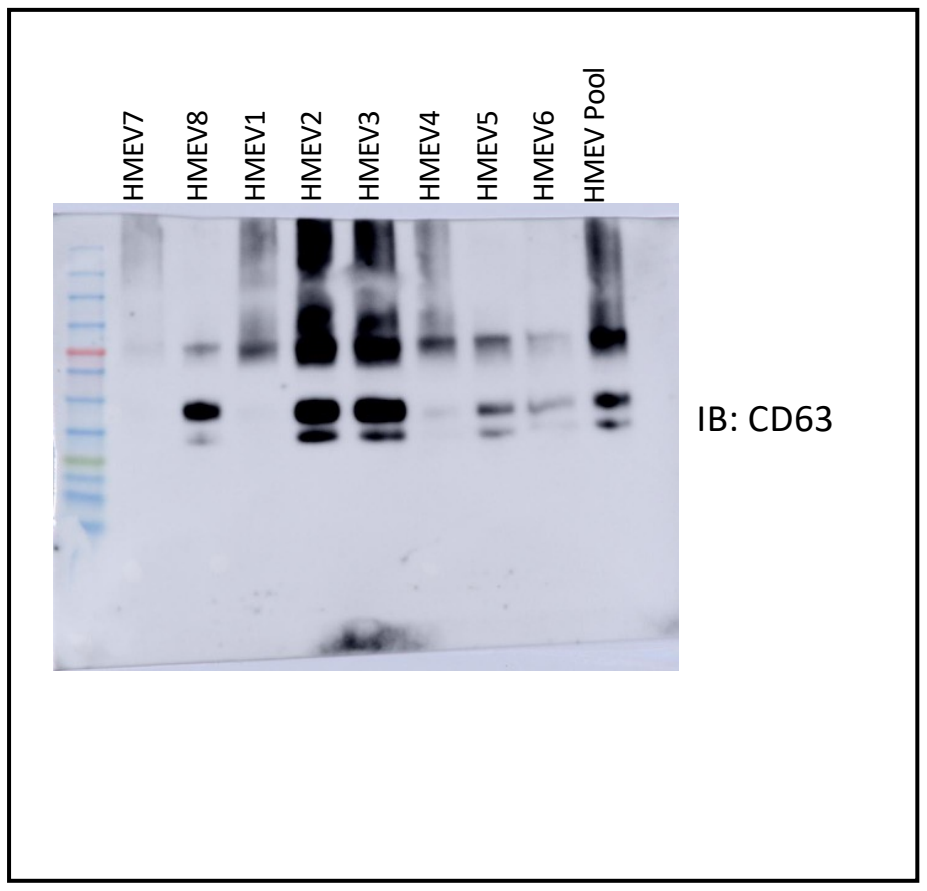
HMEV7
HMEV8
HMEV1
HMEV2
HMEV3
HMEV4
HMEV5
HMEV6
HMEV Pool
IB: CD63

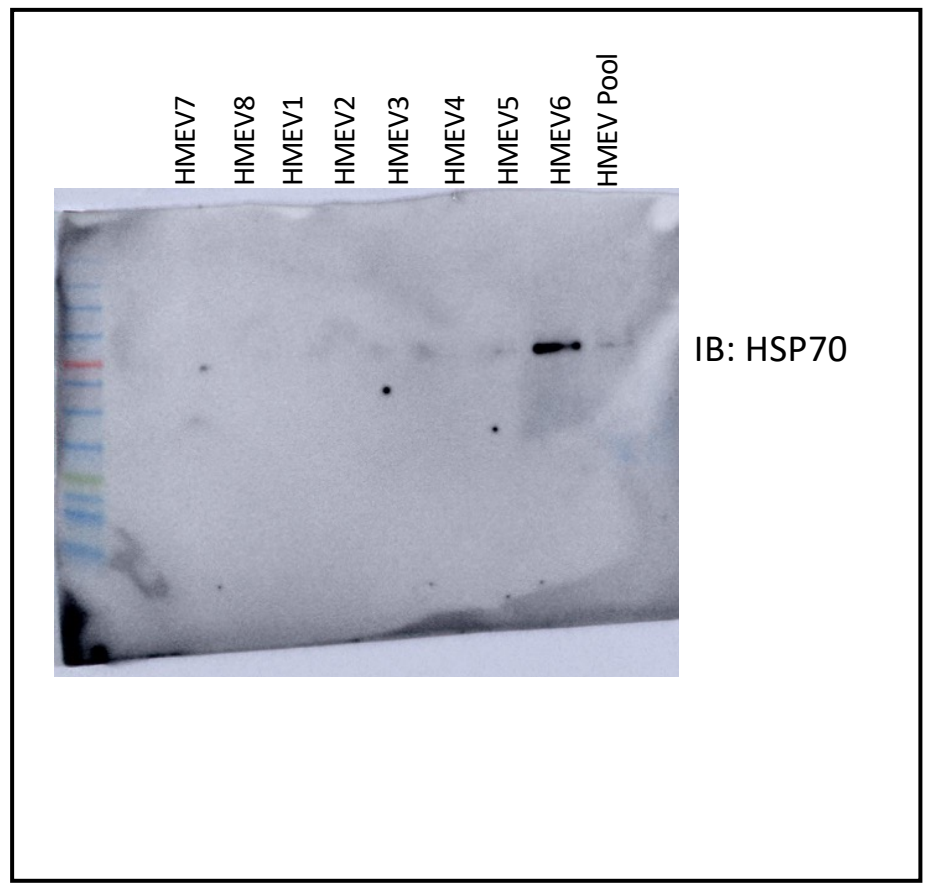
HMEV7
HMEV8
HMEV1
HMEV2
HMEV3
HMEV4
HMEV5
HMEV6
HMEV Pool
IB: HSP70

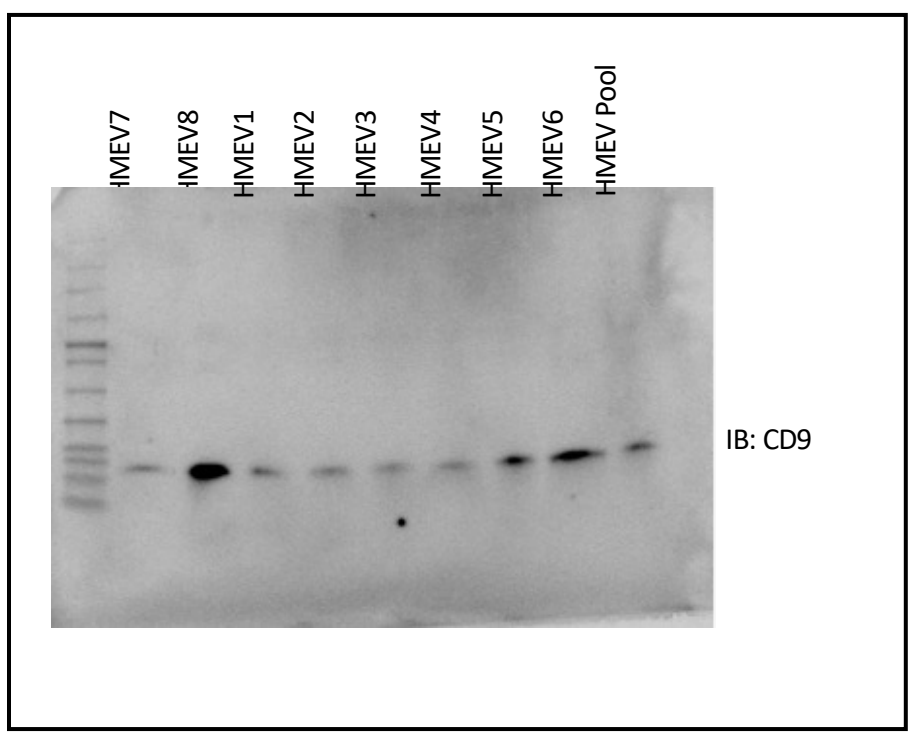
HMEV7
HMEV8
HMEV1
HMEV2
HMEV3
HMEV4
HMEV5
HMEV6
HMEV Pool
IB: CD9

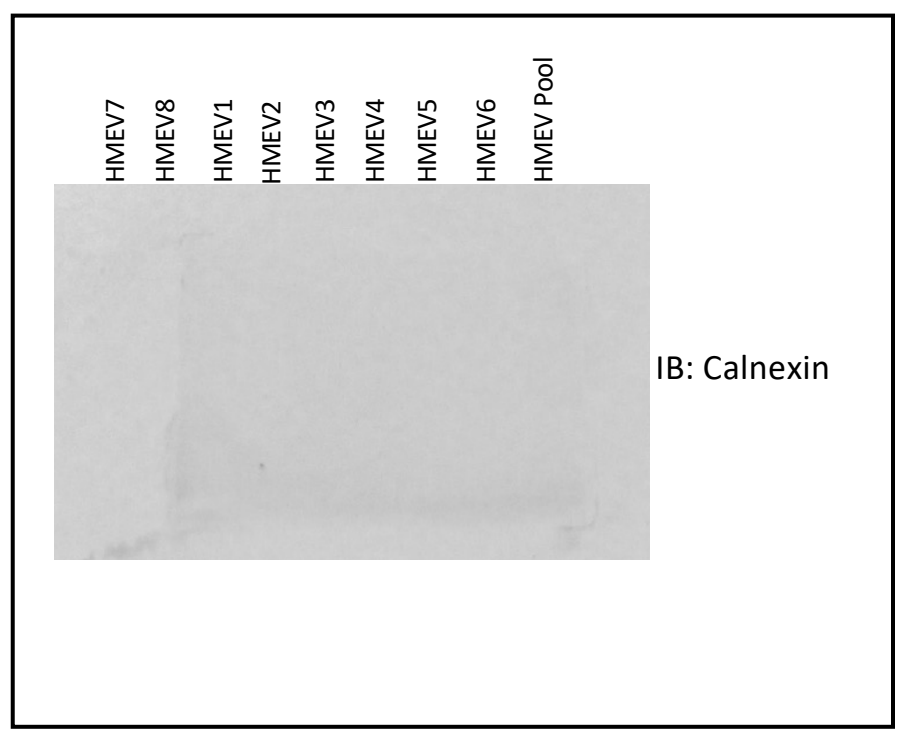
HMEV7
HMEV8
HMEV1
HMEV2
HMEV3
HMEV4
HMEV5
HMEV6
HMEV Pool
IB: Calnexin
